# Oncologic Safety of Local Excision Compared With Total Mesorectal Excision for ypT0-T1 Rectal Cancer: A Propensity Score Analysis

**DOI:** 10.1097/MD.0000000000003718

**Published:** 2016-05-20

**Authors:** Sung Min Jung, Chang Sik Yu, In Ja Park, Tae Won Kim, Jong Hoon Kim, Yong Sik Yoon, Seok-Byung Lim, Jin Cheon Kim

**Affiliations:** From the Department of Colon and Rectal Surgery (SMJ, CSY, IJP, YSY, SBL, JCK), Department of Oncology (TWK), and Department of Radiation Oncology (JHK), University of Ulsan College of Medicine, Asan Medical Center, Seoul, Republic of Korea.

## Abstract

Good oncologic outcomes, demonstrated by a complete pathologic response after preoperative chemoradiotherapy (PCRT), have led to local excision (LE) in selected patients with rectal cancer. We evaluated the oncologic safety of LE compared with total mesorectal excision (TME) in patients with ypT0-T1 rectal cancer.

A retrospective review of 304 patients who underwent PCRT, followed by LE or TME, for ypT0-T1 rectal cancer was performed. Propensity scores were computed and used to match groups (LE:TME = 1:1), and analysis of disease-free survival (DFS) and overall survival (OS) was made by comparing patients who underwent LE or TME. Prognostic factors of relapse were analyzed for all patients.

Tumor categories were ypT0 in 25 (61.9%) cases, ypTis in 6 (14.3%) cases, and ypT1 in 11 (26.2%) cases for the LE group, and ypT0 in 28 (66.7%) cases, ypTis in 4 (9.5%) cases, and ypT1 in 10 (23.8%) cases for the matched TME patients. There was no significant difference between the matched LE and TME groups in relapse (4.8% and 7.14%, respectively; *P* = 0.646), 5-year DFS (95.2% vs 91.6%; *P* = 0.33) and 5-year OS (96.6% vs 88.0%; *P* = 0.238). In the multivariate Cox regression analysis, tumor distance from the anal verge (hazard ratio [HR] = 0.78; 95% confidence interval (CI) = 0.616–0.992) and the tumor grade (HR = 4.29; 95% CI = 1.430–12.886) were significantly associated with the recurrence risk.

LE results in oncologic outcomes that are comparable to those achieved by TME in selected patients with ypT0-T1 rectal cancer after PCRT.

## Abstract 2

Supplemental Digital Content is available in the text

## INTRODUCTION

Total mesorectal excision (TME) has improved oncologic outcomes.^1^ The addition of preoperative chemoradiotherapy (PCRT) to TME effectively improves local tumor control, and is the standard treatment in advanced rectal cancer.^2–4^ However, TME entails significant complications such as perioperative morbidity associated with major surgery, anastomotic leakage, sexual and urinary dysfunction, and defecatory dysfunction.^5–7^ Furthermore, abdominoperineal resection (APR) involves a permanent stoma and low anterior resection (LAR) also often involves a temporary stoma. Surgical decision-making strategies require careful consideration of the patient's quality of life, constitutional risk factors for major surgery, and oncologic outcomes. Alternative approaches to preserve anorectal function have resulted in increased interest in local excision (LE).

The indication and oncologic safety of LE following PCRT in advanced rectal cancer has been the subject of much debate due to concerns about inadequate treatment. Following PCRT in advanced rectal cancer, LE may theoretically result in good oncologic outcomes particularly in patients demonstrating a complete pathologic response (CR).^8,9^

Several authors have reported the feasibility of performing LE following PCRT, with the majority focusing on clinical T2-3 rectal cancer or pathologic CR.^10–17^ The oncologic safety of pathologic early T status after PCRT with regard to LE has not yet been established. Data on the long-term outcomes of pathologic results and type of surgical treatment in patients with advanced rectal cancer treated with LE or TME after PCRT are required.

The aim of this study was to evaluate the oncologic outcomes of early ypT stage (ypT0-T1) in patients with mid to low rectal cancer after PCRT via a comparison of LE and TME.

## MATERIALS AND METHODS

### Patients

A total of 304 patients who underwent LE or TME between January 2006 and December 2011 were included. Eligibility criteria included patients diagnosed as ypT0-T1 on pathologic examination, completion of PCRT and surgery, location of rectal lesions in the middle to distal rectum within 10 cm from the anal verge, no distant metastasis, and no concurrent malignancy. Prior to PCRT, all patients underwent colonoscopy with biopsy. Primary tumor and nodal staging were evaluated by rectal magnetic resonance imaging (MRI) and transrectal ultrasound (TRUS). Chest, abdominopelvic computed tomography (CT) and positron emission tomography–CT were used to identify distant metastasis.

### PCRT and Surgery

All patients received PCRT according to preoperative sequential treatment protocols. Local irradiation (44–45 Gy) was delivered to the entire pelvis at a fraction dose of 180 to 200 cGy/d administered 5 d/wk, with a total of 22 to 25 fractions. This was selectively followed by a 5.4 to 6.0 Gy boost to the primary tumor (3 fractions over 3 days) and total dose to primary tumor was 50 to 50.4 Gy. The concurrent chemotherapeutic regimen was oral capecitabine (825 mg/m^2^) administered twice daily during radiotherapy or 2 cycles of 5-fluorouracil (5-FU, 375 mg/m^2^ per d) bolus with leucovorin (20 mg/m^2^ per d) for 3 days during the 1st and 5th week of radiotherapy. Rectal MRI and TRUS were repeatedly performed for restaging of the main lesion after PCRT. In patients with the size of the tumor to be almost disappeared on these imaging tools and digital rectal examination, sigmoidoscopy was performed to evaluate the state of the mucosal lesion. Between 6 and 8 weeks following completion of PCRT, all patients underwent surgery. The remaining main lesion and scar tissue were excised with at least a 1 cm margin and full depth of the rectal wall. The surgical procedures that were performed included transanal excision (TAE), transanal minimally invasive surgery (TAMIS), APR, and LAR. The patients in cases of a good clinical response (complete regression [CR] or near CR) after PCRT determine method of operation (LE vs TME) under sufficient information about the advantages and disadvantages of each approach. The reasons why patients chose LE included the refusal of APR, personal preference for less invasive surgery because of their medical comorbidity and fear of radical surgery.

### Clinicopathologic Factors

Evaluation of the tumor response to chemoradiation therapy in the excised specimen was performed using the tumor regression grading system by Dworak et al.^18^ Tumors were staged pathologically according to previously published guidelines.^19^

All patients underwent a standardized postoperative follow-up every 3 to 6 months for the first 2 years, every 6 months for the following 5 years, and annually thereafter. At each follow-up, laboratory tests were performed including analysis of CEA levels and an abdominopelvic CT scan was obtained. Chest X-ray or chest CT was performed alternately every 6 months. Colonoscopy was performed within 1 year after surgery, and then once every 2 to 3 years. In patients who underwent LE, an additional sigmoidoscopy was performed every 6 months for 2 years and then yearly for up to 5 years. Data were extracted from a prospectively maintained rectal cancer database. The study was approved by the Institutional Review Board of Asan Medical Center (No. 2015-0529).

### Statistical Analysis

**FIGURE 1:**
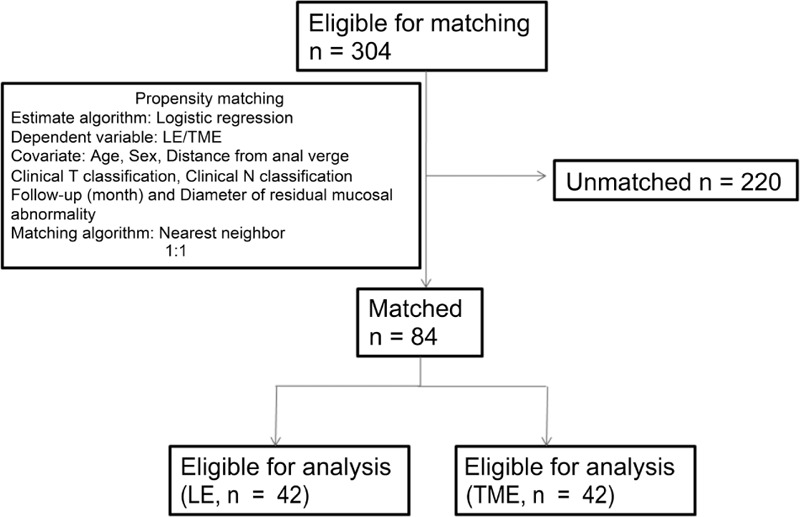
Flow diagram of the propensity matching and eligibility of patients.

Propensity scores were generated using the R package “Match It” (The Comprehensive R Archive Network: http://cran.r-project.org). To improved balance on covariates, we excluded units outside the area of common support. More detail is shown in Figure 1. Group comparisons for before and after matching were performed using the chi-squared test or Fisher exact test for categorical variable and Student *t* test for continuous variable (Table 1).

**TABLE 1:**
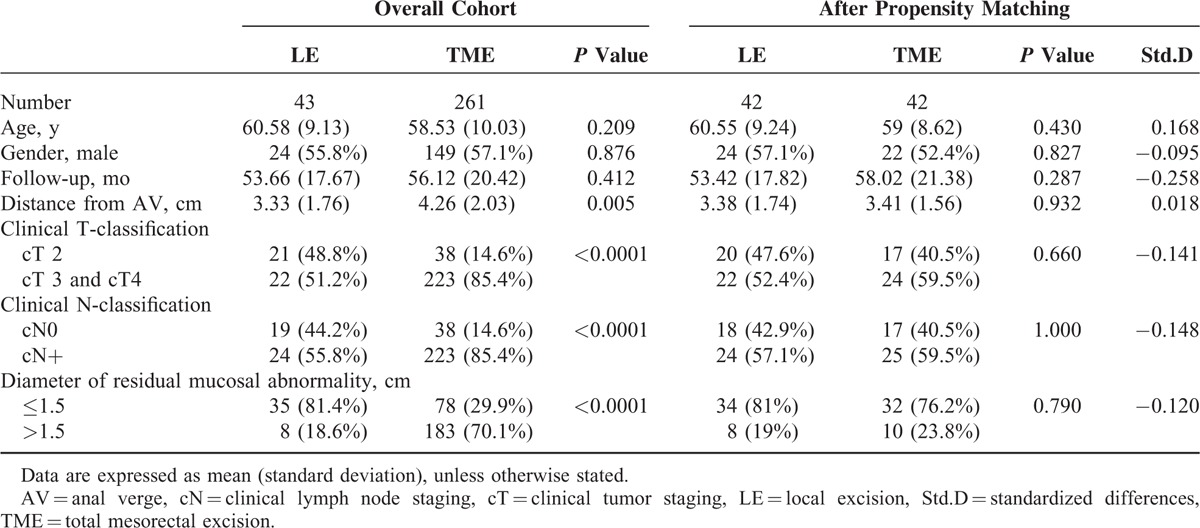
Baseline Characteristics of Patients Treated With Local Excision or Total Mesorectal Excision Following Preoperative Chemoradiotherapy

Kaplan–Meier survival analysis was used to determine the 5-year overall survival (OS) and disease-free survival (DFS), and differences between the subgroups were compared using the log-rank test. Multivariate Cox proportional hazards regression analysis of the recurrence was performed when the *P* value was <0.1 in univariate analysis by adjusting the confounder: age, gender, distance from anal verge, or tumor grade.

A *P* value <0.05 was considered statistically significant. Statistical analyses were conducted using R version 3.1.2 and SPSS for Windows, version 20.0 (IBM SPSS, IBM Corporation, Armonk, NY).

## RESULTS

### Patient Population and Tumor Characteristics

Identification of ypT0-T1 rectal adenocarcinoma was confirmed in 304 patients treated with TME or LE. TME surgery was performed in 261 patients (86%) using open approach in 229, laparoscopic in 20, and robotic in 9 patients (LAR, 199 [76.2%]; APR, 62 [23.8%]), and LE was performed in 43 patients (14%) (TAE, 38, TAMIS, 5). The mean age was 58.8 years (range, 26–82 years). Of the patients, 173 were male (57%). Clinical stage of T2 disease was observed in 59 (19%) patients, cT3 in 219 (72%) patients, and cT4 in 26 patients (9%). The median distance of the tumor from the anal verge was 4.1 cm (range, 0–10 cm). Lymph node metastasis was detected in 23 patients (8.8%) of TME group, 7.3% (13 in 176 patients) in ypT0, 4.8% (1 in 21 patients) in ypTis, and 14.1% (9 in 64 patients) in ypT1.

After propensity score matching, 84 patients (LE, 42; TME, 42) were selected for analysis (Figure 1). Median follow-up time for the matched ypT0-1 cohort was 56 months (interquartile range [IQR], 43–98 months) and was not different between the LE and TME groups (*P* = 0.287). The median age was 60 years (IQR, 52–66 years). The baseline characteristics according to the surgical procedures of the patients and the propensity matched groups are depicted in Table 1. Significant differences were found in the distance from anal verge, clinical T classification, clinical N classification, and diameter of residual mucosal abnormality between the LE and TME groups before propensity matching. After matching on the propensity score, no significant difference was observed between the LE and TME groups. The rates of lymph node metastasis in matched TME group were 7.1%. Significant differences were found for the postoperative management between LE and TME, number of patients receiving transfusion, duration of hospital stay, and adjuvant chemotherapy in Table 2.

**TABLE 2:**
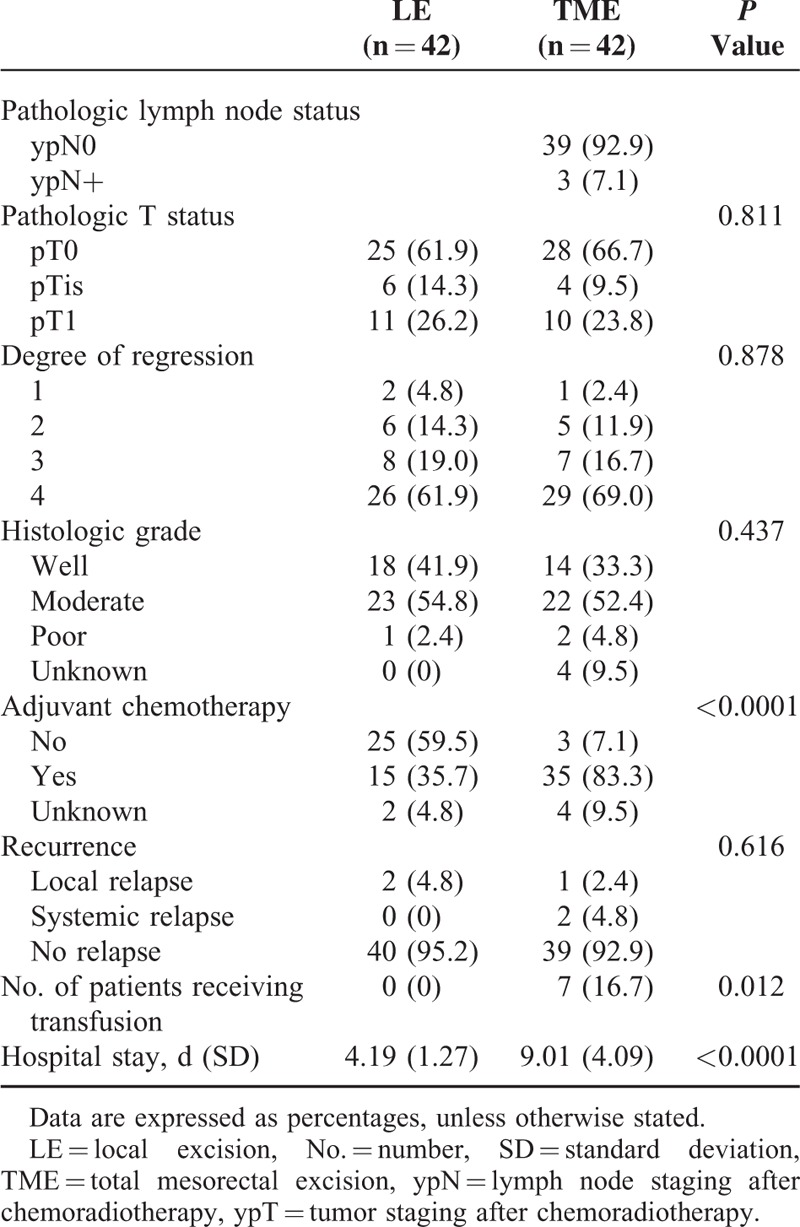
Perioperative and Pathologic Outcome

### Recurrence and Survival

**FIGURE 2:**
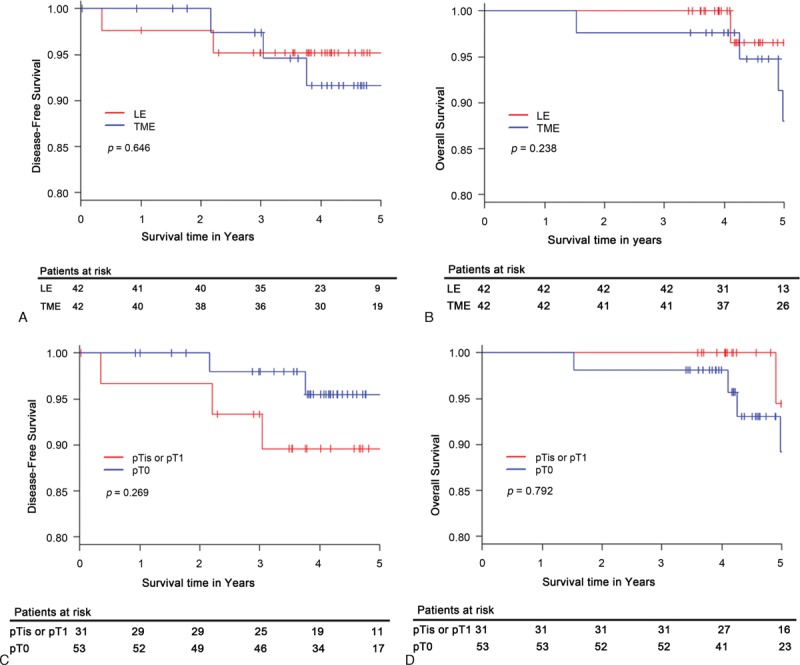
Kaplan–Meier curve according to surgical methods for matched groups (LE, n = 42; TME, n = 42): (A) disease-free survival and (B) overall survival. And survival curve for ypT0 (n = 53) and ypTis–ypT1 (n = 31) groups: (C) disease-free survival and (D) overall survival. LE = local excision, TME = total mesorectal excision.

In the matched LE group, 1 case of local relapse was observed and APR was performed. The other patient presented local relapse and underwent LAR, but developed a liver metastasis. In the TME group, 1 case of local relapse and 2 cases of systemic relapses were observed. There was no significant difference between the LE and TME groups in terms of locoregional relapse (4.8% and 2.4%, respectively; *P* = 1.0), in systemic relapse (0% and 4.8%, respectively; *P* = 0.494), in the 5-year DFS (95.2% vs 91.63%, respectively; *P* = 0.646), and in the 5-year OS (96.6% vs 88.0%; *P* = 0.238) (Figure 2A and B). In the matched cohort, no significant difference between the ypT0 and ypTis-T1 groups was found in the 5-year DFS (95.5% vs 89.6%, respectively; *P* = 0.269) and the 5-year OS (89.2% vs 94.4%; *P* = 0.792) (Figure 2C and D).

In ypT0-T1 patients, 23 were diagnosed with locoregional or systemic relapse (Supplementary Table 1). Univariate analysis for the identification of predictors of local and systemic relapse in ypT0-T1 patients revealed 2 factors that were associated with locoregional and systemic relapse (Table 3). When performing multivariate Cox regression analysis, the distance from the anal verge (hazard ratio [HR] = 0.781; 95% confidence interval [CI] = 0.616–0.992) and the tumor grade (HR = 4.293; 95% CI = 1.1430–12.886) were found to be independent predictors for overall recurrence.

**TABLE 3:**
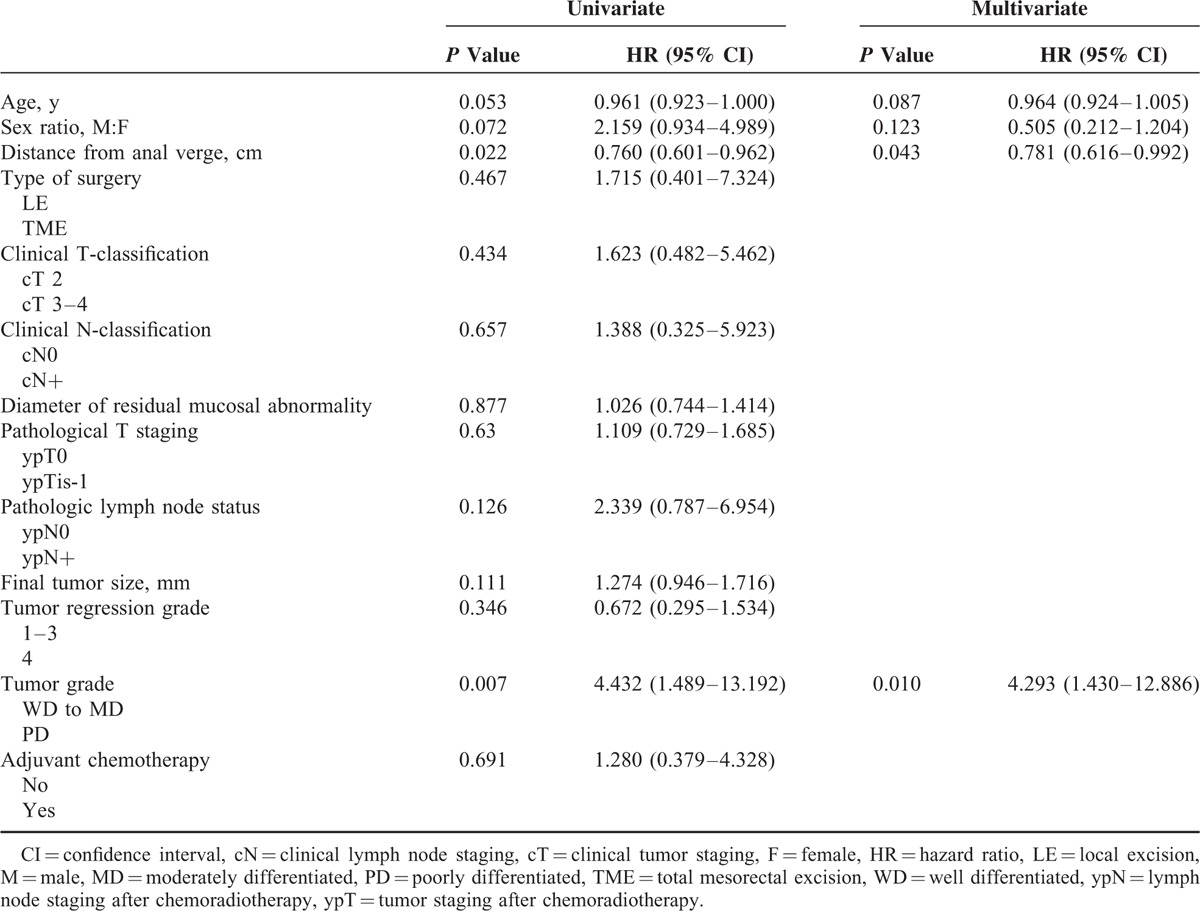
Predictors of Local and Systemic Relapse in the ypT0-1 Cohort (n = 304)

## DISCUSSION

Current improvements in oncologic outcomes of patients with advanced rectal cancer who achieved a good response after PCRT have increased the appeal of LE as a less aggressive alternative treatment. LE has been shown to lead to comparable outcomes to those of TME in selected cases.^11,17^ Considering the inability of lymph node dissection and concern of the high local recurrence rates, treatment of advanced rectal cancer with LE is rather controversial. Therefore, it is essential to select patients who represent ideal candidates with a minimal risk of local and systemic relapse for LE treatment.

In the present study, patients with ypT0-T1 rectal tumors had an excellent prognosis. The type of surgery did not appear to determine prognosis in ypT0-T1 patients. Propensity score analysis demonstrated a similar 5-year OS and 5-year DFS rate following LE or TME in patients diagnosed with ypT0-T1 after PCRT. In the LE group, local recurrence was observed only in patients with the ypT1 stage. However, in the propensity analysis, there was no significant difference in local recurrence between LE and TME in patients with ypT1. Moreover, between ypT0 and ypTis-T1, DFS and OS did not differ significantly in all matched patients with ypT0-T1.

As regional lymph node dissections are not performed using LE, the probability of lymph node metastasis is an important consideration in decision making for LE. As tumor regression after PCRT in primary tumors is usually accompanied by tumor regression in mesorectal metastatic lymph nodes; many authors have reported a lower rate of lymph node metastasis in good responders of primary tumor after PCRT.^16,20–24^ Borschitz et al^13^ suggested that LE in good responders to PCRT have relatively low rates of nodal metastasis and local recurrence. The correlation between the risk of lymph node metastasis and ypT status was also previously demonstrated.^25–27^ In the present study, the rate of lymph node metastasis in the TME group in the matched cohort was also found to be low, and which made us assume that the rate in the LE group may be lower or at least similar than that of the TME group due to the strict evaluation of lymph node metastasis prior to surgery. The GRCCA 2 study which randomized good clinical responders (residual tumor <2 cm) after PCRT between LE and TME shows extremely low rates of LN metastasis in ypT0-1 (0%) compared with ypT2-3 (15%).^28^

There is considerable debate over whether or not LE should be performed following PCRT in advanced rectal cancer patients. Previous studies that investigated the feasibility of LE primarily focused on clinical T2-3 rectal cancer, and favorable outcomes were mainly demonstrated for selected cases, especially for those downstaged to ypT0.^10–14^ These studies could not reflect the regression response after PCRT which was the important prognostic factor. A recent randomized trial in patients with clinical T2 rectal cancer, especially in patients with a tumor diameter no >3 cm and a histologic grade of G1-2, showed an equivalent DFS for TME and LE.^17^ In this study, local and systemic relapse occurred only in low or nonresponders to PCRT, and suggested that the degree of response to PCRT might be used as a factor in decision making to perform LE. Their results demonstrated the outcomes of patients who had undergone LE were similar to those of TME, and that LE can be clinically indicated in certain cases. However, it is difficult to be generalized from this study, because the indication for LE did not consider the responses to PCRT and did not include patients with locally advanced (clinical stage II–III) rectal cancer.

Because of the good prognosis of patients with a good bowel wall response (ypT0-T1) to PCRT with TME, organ-preserving approaches must be compared with radical resection in terms of oncologic safety. This study demonstrated excellent oncologic outcomes after LE compared with that of TME in these patients. In addition, there were no significant differences in DFS between patients with ypT0 disease and those with ypTis-T1 disease in LE group. This demonstrated that complete resection of primary tumor sites plays a potential curative role even in patients with a residual tumor on the rectal wall after PCRT. Unfortunately, there remain many difficulties in predicting the remission grade prior to surgery. The sensitivity and specificity of MRI restaging is not reliable.^29^ As a result, we occasionally observed residual tumors that did not correlate with clinical CR. If this discrepancy is observed following LE, an immediate decision should be made regarding the feasibility of subsequent TME surgery. The good oncologic outcomes of LE in patients with ypTis-T1 in our study may reduce concerns about the need for subsequent TME surgery in these patients with a residual tumor on the rectal wall after PCRT.

In a previous study comparing LE with TME, only patients with ypT2 were found to develop local recurrence.^30^ Considering the possibility of lymph node metastasis and poor outcomes after LE,^31^ we believe that LE of ypT2 tumors with curative intent has a negligible role, and that immediate salvage surgery, such as LAR or APR should be performed if a residual tumor within the bowel wall up to ypT2 is confirmed after LE. Therefore, we did not compare oncologic outcomes in ypT2 patients.

In the multivariate analysis, the risk factors for recurrence in the ypT0-T1 cohort were the pathologic tumor grade and distance from the anal verge. The presence of a metastatic lymph node is an important prognostic factor for recurrence,^9^ but we did not find a statistically significant association between a metastatic lymph node and recurrence in TME group of this study. This demonstrates that TME after PCRT has a potential curative role in good responders to PCRT, even among patients with proven metastatic lymph nodes. Hence LE could be performed with a low risk of recurrence when major remission, including a low probability of lymph node metastasis, is anticipated on re-staging despite of a final pathologic result of ypTis-T1.

There were several limitations in this study. First, this study is a retrospective study; in order to prove our hypothesis, it is extremely difficult and ethically questionable to perform a randomized trial. Second, it is difficult to compare the standard treatment, TME, to the alternative treatment, LE, without bias. Although we attempted to reduce selection bias by using propensity score matching, it was not possible to completely eliminate subjective judgments affecting the determination of the therapeutic method. Despite similar pathologic statuses, adjuvant chemotherapy was conducted more frequently in the TME group. Nevertheless, there was no difference in survival between groups. Third, as this was a single institution and small size study, the findings are rather difficult to generalize. Because LE combined with PCRT should be cautiously applied in selected patients, our study participant was small. Large, multicenter studies are required to overcome this problem. Finally, the exclusion of patients in the propensity score-matched analysis resulted in a loss of statistical power.

## CONCLUSIONS

Comparable oncologic results to those obtained after TME was achieved by LE in patients with ypT0-T1 advanced rectal cancer after PCRT. This result demonstrates that the LE could be an optional treatment for the patients achieved clinical good response after PCRT and immediate salvage TME should be considered in cases of ypT2-3. A larger sample sized study and randomized trial will be necessary to verify this promising result.

## Supplementary Material

Supplemental Digital Content

## References

[1] Heald RJ Ryall RD Recurrence and survival after total mesorectal excision for rectal cancer. *Lancet* 1986; 1:1479–1482. 2425199 10.1016/s0140-6736(86)91510-2

[2] Sauer R Becker H Hohenberger W Preoperative versus postoperative chemoradiotherapy for rectal cancer. *N Engl J Med* 2004; 351:1731–1740. 15496622 10.1056/NEJMoa040694

[3] Bosset JF Collette L Calais G Chemotherapy with preoperative radiotherapy in rectal cancer. *N Engl J Med* 2006; 355:1114–1123. 16971718 10.1056/NEJMoa060829

[4] Bianchi PP Petz W Luca F Laparoscopic and robotic total mesorectal excision in the treatment of rectal cancer. Brief review and personal remarks. *Front Oncol* 2014; 4:98. 24834429 10.3389/fonc.2014.00098 PMC4018567

[5] Chessin DB Enker W Cohen AM Complications after preoperative combined modality therapy and radical resection of locally advanced rectal cancer: a 14-year experience from a specialty service. *J Am Coll Surg* 2005; 200:876–882.discussion 882–874. 15922198 10.1016/j.jamcollsurg.2005.02.027

[6] Havenga K Enker WE McDermott K Male and female sexual and urinary function after total mesorectal excision with autonomic nerve preservation for carcinoma of the rectum. *J Am Coll Surg* 1996; 182:495–502. 8646349

[7] Peeters KCMJ Tollenaar RAE Marijnen CAM Risk factors for anastomotic failure after total mesorectal excision of rectal cancer. *Br J Surg* 2005; 92:211–216. 15584062 10.1002/bjs.4806

[8] Maas M Nelemans PJ Valentini V Long-term outcome in patients with a pathological complete response after chemoradiation for rectal cancer: a pooled analysis of individual patient data. *Lancet Oncol* 2010; 11:835–844. 20692872 10.1016/S1470-2045(10)70172-8

[9] Lim SB Yu CS Hong YS Long-term outcomes in patients with locally advanced rectal cancer treated with preoperative chemoradiation followed by curative surgical resection. *J Surg Oncol* 2012; 106:659–666. 22674581 10.1002/jso.23181

[10] Bonnen M Crane C Vauthey JN Long-term results using local excision after preoperative chemoradiation among selected T3 rectal cancer patients. *Int J Radiat Oncol Biol Phys* 2004; 60:1098–1105. 15519780 10.1016/j.ijrobp.2004.04.062

[11] Yu CS Yun HR Shin EJ Local excision after neoadjuvant chemoradiation therapy in advanced rectal cancer: a national multicenter analysis. *Am J Surg* 2013; 206:482–487. 23849272 10.1016/j.amjsurg.2013.01.042

[12] Nair RM Siegel EM Chen DT Long-term results of transanal excision after neoadjuvant chemoradiation for T2 and T3 adenocarcinomas of the rectum. *J Gastrointest Surg* 2008; 12:1797–1805. 18709419 10.1007/s11605-008-0647-z

[13] Borschitz T Wachtlin D Mohler M Neoadjuvant chemoradiation and local excision for T2-3 rectal cancer. *Ann Surg Oncol* 2008; 15:712–720. 18163173 10.1245/s10434-007-9732-x

[14] Noh JM Park W Kim JS Outcome of local excision following preoperative chemoradiotherapy for clinically T2 distal rectal cancer: a multicenter retrospective study (KROG 12-06). *Cancer Res Treat* 2014; 46:243–249. 25038759 10.4143/crt.2014.46.3.243 PMC4132444

[15] Pucciarelli S De Paoli A Guerrieri M Local excision after preoperative chemoradiotherapy for rectal cancer: results of a multicenter phase II clinical trial. *Dis Colon Rectum* 2013; 56:1349–1356. 24201388 10.1097/DCR.0b013e3182a2303e

[16] Kundel Y Brenner R Purim O Is local excision after complete pathological response to neoadjuvant chemoradiation for rectal cancer an acceptable treatment option? *Dis Colon Rectum* 2010; 53:1624–1631. 21178856 10.1007/DCR.0b013e3181f5b64d

[17] Lezoche E Baldarelli M Lezoche G Randomized clinical trial of endoluminal locoregional resection versus laparoscopic total mesorectal excision for T2 rectal cancer after neoadjuvant therapy. *Br J Surg* 2012; 99:1211–1218. 22864880 10.1002/bjs.8821

[18] Dworak O Keilholz L Hoffmann A Pathological features of rectal cancer after preoperative radiochemotherapy. *Int J Colorectal Dis* 1997; 12:19–23. 9112145 10.1007/s003840050072

[19] Edge S Byrd DR Compton CC AJCC Cancer Staging Manual. 7th ed. New York: Springer; 2010.

[20] Coco C Manno A Mattana C The role of local excision in rectal cancer after complete response to neoadjuvant treatment. *Surg Oncol* 2007; 16 suppl 1:S101–S104. 18023178 10.1016/j.suronc.2007.10.008

[21] Caricato M Ausania F De Dominicis E Tumor regression in mesorectal lymphnodes after neoadjuvant chemoradiation for rectal cancer. *Eur J Surg Oncol* 2007; 33:724–728. 17336482 10.1016/j.ejso.2007.01.023

[22] Hughes R Glynne-Jones R Grainger J Can pathological complete response in the primary tumour following pre-operative pelvic chemoradiotherapy for T3–T4 rectal cancer predict for sterilisation of pelvic lymph nodes, a low risk of local recurrence and the appropriateness of local excision? *Int J Colorectal Dis* 2006; 21:11–17. 15864605 10.1007/s00384-005-0749-y

[23] Pucciarelli S Capirci C Emanuele U Relationship between pathologic T-stage and nodal metastasis after preoperative chemoradiotherapy for locally advanced rectal cancer. *Ann Surg Oncol* 2005; 12:111–116. 15827790 10.1245/ASO.2005.03.044

[24] Bedrosian I Rodriguez-Bigas MA Feig B Predicting the node-negative mesorectum after preoperative chemoradiation for locally advanced rectal carcinoma. *J Gastrointest Surg* 2004; 8:56–62. 14746836 10.1016/j.gassur.2003.09.019

[25] Bujko K Nowacki MP Nasierowska-Guttmejer A Prediction of mesorectal nodal metastases after chemoradiation for rectal cancer: results of a randomised trial: implication for subsequent local excision. *Radiother Oncol* 2005; 76:234–240. 16273666 10.1016/j.radonc.2005.04.004

[26] Mignanelli ED de Campos-Lobato LF Stocchi L Downstaging after chemoradiotherapy for locally advanced rectal cancer: is there more (tumor) than meets the eye? *Dis Colon Rectum* 2010; 53:251–256. 20173469 10.1007/DCR.0b013e3181bcd3cc

[27] Tranchart H Lefèvre JH Svrcek M What is the incidence of metastatic lymph node involvement after significant pathologic response of primary tumor following neoadjuvant treatment for locally advanced rectal cancer? *Ann Surg Oncol* 2013; 20:1551–1559. 23188545 10.1245/s10434-012-2773-9

[28] Vendrely V Rullier E Rouanet P Local excision versus total mesorectal excision in patients with good response after neoadjuvant radiochemotherapy for T2–T3 low rectal cancer: preliminary results of the GRECCAR 2 randomized phase 3 trial. *Int J Radiat Oncol Biol Phys* 2014; 1:S20.

[29] Moreno CC Sullivan PS Kalb BT Magnetic resonance imaging of rectal cancer: staging and restaging evaluation. *Abdom Imaging* 2015; 40:2613–2629. 25759246 10.1007/s00261-015-0394-z

[30] Quah HM Chou JF Gonen M Pathologic stage is most prognostic of disease-free survival in locally advanced rectal cancer patients after preoperative chemoradiation. *Cancer* 2008; 113:57–64. 18442099 10.1002/cncr.23516

[31] You YN Roses RE Chang GJ Multimodality salvage of recurrent disease after local excision for rectal cancer. *Dis Colon Rectum* 2012; 55:1213–1219. 23135578 10.1097/DCR.0b013e318270837f

