# Supplementary material for: Oncologic Safety of Local Excision Compared With Total Mesorectal Excision for ypT0-T1 Rectal Cancer: A Propensity Score Analysis
Source: Medicine (Baltimore). 2016 May 20;95(20):e3718. doi: 10.1097/MD.0000000000003718 (PMC4902432; doi:10.1097/MD.0000000000003718)

**Supplementary TABLE 1**Clinicopathologic characteristics according to disease recurrence

|  |  |  | CRT |  |  |  | Primary rectal cancer | | |  |  |  | Recurrence |  |  |
| --- | --- | --- | --- | --- | --- | --- | --- | --- | --- | --- | --- | --- | --- | --- | --- |
| Group | OP Type | Gender/Age | CRT | Response | AdjCTx |  | AV (cm) | Grade | cT | ypT | ypN |  | Site | DFS (mon) | Status |
| Matched | LE | F/51 | 44/Xeloda | Near total | No |  | 5 | MD | 3 | ypT1 |  |  | Local | 4 | Alive |
| Matched | LE | M/59 | 50/FL#2 | Moderate | No |  | 3 | MD | 2 | ypT1 |  |  | Regional LN, Liver | 26 | Alive |
| Matched | TME | F/34 | 50/xeloda | Near total | FL#4 |  | 3 | Unknown | 3 | ypT1 | Negative |  | Local | 36 | Death |
| Matched | TME | F/66 | 50/xeloda | Total | Xeloda |  | 2 | Unknown | 3 | ypT0 | Negative |  | Lung | 26 | Death |
| Matched | TME | F/54 | 50/FL#2 | Total | FL#4 |  | 0 | MD | 3 | ypT0 | Positive |  | Liver, Lung | 45 | Alive |
| None-Matched | TME | M/60 | 50/xeloda | Total | Xeloda |  | 1 | PD | 4 | ypT0 | Negative |  | Regional LN | 6 | Alive |
| None-Matched | TME | F/53 | 50.6/FL#2 | Total | FL#4 |  | 1 | WD | 3 | ypT0 | Negative |  | Lung | 17 | Alive |
| None-Matched | TME | F/59 | 50.4/xeloda | Near total | Xeloda |  | 3 | WD | 2 | ypTis | Negative |  | Lung | 20 | Death |
| None-Matched | TME | F/68 | 50/xeloda | Near total | Xeloda |  | 5 | WD | 3 | ypT1 | Negative |  | Liver | 27 | Death |
| None-Matched | TME | M/60 | 50/xeloda | Total | Xeloda |  | 1 | WD | 4 | ypT0 | Negative |  | Lung | 34 | Alive |
| None-Matched | TME | F/38 | 50/xeloda | Total | Xeloda |  | 4 | MD | 3 | ypT0 | Negative |  | Lung | 6 | Alive |
| None-Matched | TME | M/50 | 48/xeloda | Total | No |  | 4 | MD | 3 | ypT0 | Negative |  | Lung | 11 | Death |
| None-Matched | TME | M/52 | 50/xeloda | Moderate | Xeloda |  | 2 | MD | 3 | ypT1 | Negative |  | Lung | 13 | Death |
| None-Matched | TME | F/68 | 50/xeloda | Near total | FL#4 |  | 3 | MD | 3 | ypT1 | Positive |  | Lung | 10 | Alive |
| None-Matched | TME | M/40 | 50/xeloda | Total | xeloda |  | 2 | MD | 3 | ypT0 | Negative |  | Lung | 37 | Alive |
| None-Matched | TME | F/55 | 50.4/SOX#4 | Total | SOX#6 |  | 4 | MD | 3 | ypT0 | Positive |  | Lung | 30 | Alive |
| None-Matched | TME | F/36 | 50/FL#2 | Total | FL#4 |  | 6 | PD | 3 | ypT0 | Negative |  | Lung | 11 | Death |
| None-Matched | TME | F/66 | 50/FLOX#3 | Moderate | FL#8 |  | 4 | MD | 2 | ypT1 | Negative |  | Peritoneal seeding | 52 | Alive |
| None-Matched | TME | M/56 | 44/FL#2 | Near total | FL#4 |  | 4 | PD | 3 | ypT0 | Negative |  | Lung, Local | 7 | Death |
| None-Matched | TME | F/51 | 50/FL#2 | Total | FL#4 |  | 5 | MD | 3 | ypT0 | Negative |  | Lung | 18 | Alive |
| None-Matched | TME | F/49 | 50/FL#2 | Total | FOLFOX#8 |  | 6 | WD | 3 | ypT0 | Positive |  | Lung | 6 | Death |
| None-Matched | TME | M/61 | 50/FL#2 | Moderate | FL#4 |  | 6 | MD | 3 | ypT1 | Positive |  | Systemic LN | 48 | Alive |
| None-Matched | TME | M/68 | 50.4/FL#2 | Total | FL#4 |  | 0 | PD | 4 | ypT0 | Negative |  | Peritoneal seeding | 13 | Death |

*LE* local excision,*TME* total mesorectal excision,*CRT* chemoradiotherapy,*AV* distance from anal verge,*cT* clinical tumor staging,*ypT* tumor staging after chemoradiotherapy,*ypN* lymph node staging after chemoradiotherapy,*LN* lymph node,*WD* well differentiated,*MD* moderately differentiated,*PD* poorly differentiated,*DFS* disease free survival,*Xeloda* capecitabine,*FL* fluorouracil/leucovorin,*SOX*tegafur/gimeracil/oteracil/oxaliplatin,*FLOX* fluorouracil/leucovorin/oxaliplatin,*FOLFOX* leucovorin/fluorouracil/oxaliplatin

Supplemental Digital Content 1.Figure that demonstrates the distribution of propensity scores with a comparison of matched and non-matched cases and controls. tiff


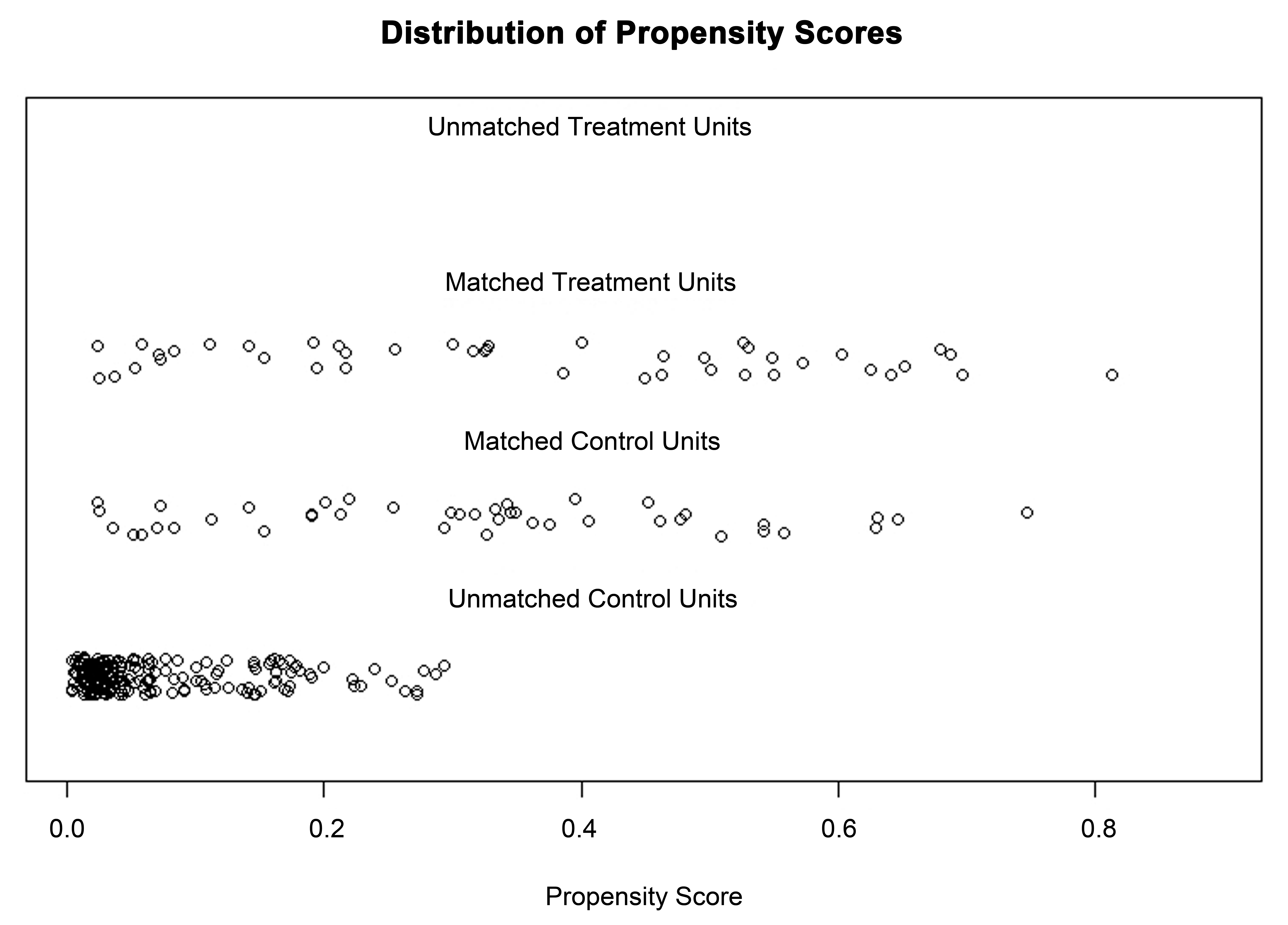


Supplemental Digital Content 2. Figure that demonstrates the histogram of propensity scores with a comparison of matched and non-matched cases. Tiff


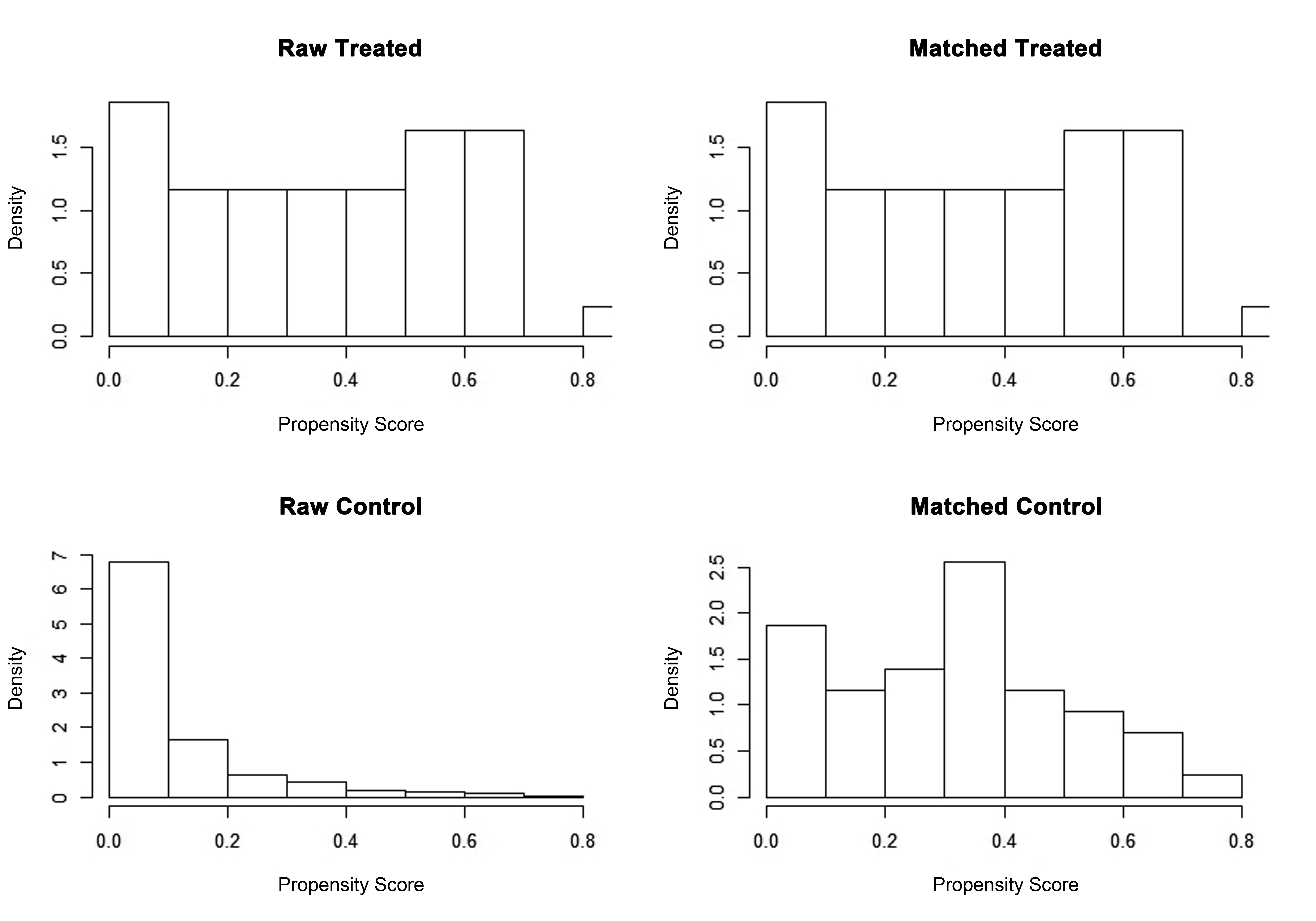

Supplement: Supplemental Digital Content [file medi-95-e3718-s001.doc]
